# Variations in chemical fingerprints and major flavonoid contents from the leaves of thirty‐one accessions of *Hibiscus sabdariffa* L.

**DOI:** 10.1002/bmc.3623

**Published:** 2015-10-28

**Authors:** Jin Wang, Xianshuang Cao, Vanessa Ferchaud, Yadong Qi, Hao Jiang, Feng Tang, Yongde Yue, Kit L. Chin

**Affiliations:** ^1^ SFA Key Laboratory of Bamboo and Rattan Science and Technology, International Centre for Bamboo and RattanNo. 8 Futong Dongdajie, Wangjing, Chaoyang DistrictBeijing100102China; ^2^ Southern University Agricultural Research and Extension CenterBaton RougeLA70813USA; ^3^ Urban Forestry Program, College of Science and AgricultureSouthern UniversityBaton RougeLA70813USA

**Keywords:** *Hibiscus sabdariffa* L, flavonoids, fingerprints, principal components analysis (PCA), LC‐Q‐TOF‐MS

## Abstract

The leaves of *Hibiscus sabdariffa* L. have been used as traditional folk medicines for treating high blood pressure and fever. There are many accessions of *H*. *sabdariffa* L. throughout the world. To assess the chemical variations of 31 different accessions of *H*. *sabdariffa* L., fingerprinting analysis and quantitation of major flavonoids were performed by high‐performance liquid chromatography (HPLC). The HPLC method was validated for linearity, sensitivity, precision, repeatability and accuracy. A quadrupole‐time‐of‐flight mass spectrometry (Q‐TOF‐MS) was applied for the characterization of major compounds. A total of 9 compounds were identified, including 6 flavonoids and 3 phenolic acids. In the fingerprint analysis, similarity analysis (SA) and principal component analysis (PCA) were used to differentiate the 31 accessions of *H. sabdariffa* L. Based on the results of PCA and SA, the samples No. 15 and 19 appeared much different from the main group. The total content of five flavonoids varied greatly among different accessions, ranging from 3.35 to 23.30 mg/g. Rutin was found to be the dominant compound and the content of rutin could contribute to chemical variations among different accessions*.* This study was helpful to understand the chemical variations between different accessions of *H. sabdariffa* L., which could be used for quality control. © 2015 The Authors Biomedical Chromatography Published by John Wiley & Sons Ltd.

**Abbreviations used**
ACN: acetonitrile
HPLC: high performance liquid chromatography
ICH: International Conference on Harmonisation
LC‐Q‐TOF‐MS: liquid chromatography‐quadrupole‐time‐of‐flight mass spectrometry
LOD: limit of detection
LOQ: limit of quantification
PA: peak area
PCA: principal component analysis
PC1: the first principal component
PC2: the second principal component
RSD: relative standard deviations
RT: retention time
SA: similarity analysis
S/N: signal‐to‐noise ratio
SUAREC: Southern University Agricultural Research and Extension Center
USDA‐ARS: United States Department of Agriculture‐Agricultural Research Service
UV: ultraviolet
WHO: World Health Organization.

## Introduction

*Hibiscus sabdariffa* L. (family: Malvaceae) is used for both food and traditional medicine (Da‐Costa‐Rocha *et al*., 2014). It is most popular for its calyces used as sour tea (Ali *et al*., 2005). In India, Africa and Mexico, infusions of the leaves or calyces are traditionally used for their diuretic, cholerectic, febrifugal and hypotensive effects (Kuo *et al*., 2012; Guardiola and Mach, 2014). Research has shown that *H. sabdariffa* L. leaves have multiple biological activities, such as antiatherosclerotic effect (Chen *et al*., 2013), anticancer activity (Lin *et al*., 2012), antioxidant and antihyperlipidemic activities (Ochani and D'Mello, 2009; Gosain *et al*., 2010; Sindi *et al*., 2014). Flavonoids and phenolic acids are considered as the major bioactive compounds in the leaves of *H. sabdariffa* L. (Chen *et al*., 2013).

Many accessions (samples of a crop variety collected at a specific location and time) of *H*. *sabdariffa* L. are widely cultivated in Africa, Asia, and America (Patel, 2014). The *H. sabdariffa* L. leaves from different countries and accessions could have different chemical constituents, which may result in the improper clinical usage under the same name. Most of the research about *H. sabdariffa* L. does not specify the origin of the variety and the crop site, making it difficult to make comparisons between the chemical profile and bioactivities of extracts obtained in different studies (Borrás‐Linares *et al*., 2015). Furthermore, the amount of bioactive compounds in *H. sabdariffa* L. leaves is an important aspect that influences their therapeutic effects. Therefore, to evaluate chemical variations of different accessions of *H. sabdariffa* L. is needed.

A strategy for clarifying the chemical variations of different accessions of *H. sabdariffa* L. consist of two aspects. One is the qualitative and quantitative analysis of several bioactive components (Jin *et al*., 2008). The other is chemical fingerprint analysis, which has been accepted by World Health Organization (WHO) (Kong *et al*., 2009; World Health Organization, 1991). At present, fingerprint analysis, based on multivariate statistical analysis, such as similarity analysis (SA) and principal component analysis (PCA), is widely applied to discriminate the medicinal plants (Tian *et al*., 2009; Xu *et al*., 2011), and fruits (Sârbu *et al*., 2012).

Flavonoids in edible and medicinal plants possess wide range of biochemical and pharmacological effects. Rutin, quercetin and its derivatives, and kaempferol and its derivatives are identified as major flavonoids in *H. sabdariffa* L. leaves (Zhen *et al*., 2016). The flavonoid content is an important factor in plant foods, which has been archived in the United States Department of Agriculture (USDA) database (U.S. Department of Agriculture, Agricultural Research Service, 2013). Therefore, flavonoids could be used as marker compounds to evaluate chemical consistency among 31 *H. sabdariffa* L. leaves. In our previous studies, high radical scavenging activity was observed in the leaves of *H. sabdariffa* L. The activity varied among different accessions (Wang *et al*., 2014). Therefore, this study was designed to profile the chemical fingerprint in the leaves of 31 *H. sabdariffa* L. accessions cultivated in United States. A Q‐TOF‐MS was carried out to identify the major fingerprint peaks*.* The chemical profiles of *H. sabdariffa* L. leaves were investigated by chemometric methods. Moreover, a reliable HPLC method was developed and validated for simultaneous determination of five flavonoids in the leaves of 31 *H. sabdariffa* L. accessions.

## Materials and methods

### Chemicals and reagents

**Figure 1:**
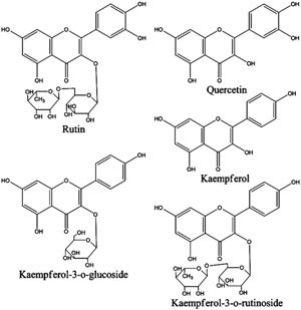
Structures of five investigated flavonoids.

HPLC‐grade acetonitrile (ACN), methanol, water and formic acid were obtained from Fisher Scientific (Fair Lawn, NJ, USA). Standards including quercetin (95% purity) and rutin (97% purity) were purchased from Acros Organics (Morris Plains, NJ, USA). Kaempferol (97% purity) was purchased from Sigma Aldrich (St. Louis, MO, USA). Kaempferol‐3‐o‐rutinoside (98% purity) and kaempferol‐3‐o‐glucoside (99% purity) were obtained from Indofine Chemicals (Hillsborough, NJ). Their chemical structures are shown in Fig. 1.

### Plant materials

A total of 31 accessions of *H. sabdariffa* L. were included in this study. Their sample identity numbers, country origins, and accession labels are listed in the Table 1. The first 25 accessions were obtained from United States Department of Agriculture‐Agricultural Research Service (USDA‐ARS) Plant Genetic Resources Conservation Unit in Griffin, Georgia and the additional 6 were collected by the Southern University Agricultural Research and Extension Center (SUAREC) Hibiscus Research Group.

**Table 1:** Thirty‐one accessions of *H. sabdariffa* L.

No.	Country (seed source)	Accession label
1	India	PI^a^‐180026
2	Cuba	PI‐207920
3	Bangladesh	PI‐256038
4	Bangladesh	PI‐256039
5	Poland	PI‐256041
6	Cuba, La Habana	PI‐265319
7	Sudan	PI‐267778
8	Nigeria	PI‐268097
9	Nigeria	PI‐268100
10	Taiwan	PI‐273388
11	Taiwan	PI‐273389
12	Taiwan	PI‐273391
13	South Africa, Transvaal	PI‐273459
14	Nigeria	PI‐274245
15	Senegal	PI‐275413
16	Ghana	PI‐286316
17	Ghana	PI‐286319
18	Thailand	PI‐365477
19	United States	PI‐468411
20	United States, Georgia	PI‐468413
21	Sudan	PI‐496717
22	Sudan	PI‐496938
23	Zambia	PI‐500725
24	Zambia	PI‐500737
25	South Africa	PI‐638933
26	Jamaica	SUAREC^b^‐1
27	Liberia	SUAREC‐2
28	Nigeria	SUAREC‐3
29	Senegal	SUAREC‐4
30	Thailand	SUAREC‐5
31	South Africa	SUAREC‐6

a PI, Plant Identification. The PI numbers were assigned by the USDA‐ARS.

b SUAREC, the accessions collected by the Southern University Agricultural Research and Extension Center.

The seeds from all the accessions were germinated in the greenhouse in March 2012. On May 14, 2012, the seedlings were transplanted to the field in the Horticultural Farm at Southern University, Baton Rouge, Louisiana, USA. The leaves were collected in June 2012 and authenticated by Dr. Kit L. Chin (Southern University Agricultural Research and Extension Center). Leaf samples were oven‐dried and ground into fine powder.

### Preparation of standard and sample solutions

Dried *H*. *sabdariffa* L. leaves powder (0.20 g) were accurately weighed and extracted with 70% aqueous methanol (20 mL) by ultrasonic‐assisted extraction (44 KHz, 70 W, Branson Ultrasonic Corporation, Danbury, CT, USA) for 30 min at room temperature(Chen *et al*., 2012; Zu *et al*., 2006). Each sample was prepared in triplicate for quantification of analytes. The sample solutions were filtered through 0.45‐µm membrane.

Stock solutions of the five analytes were prepared in methanol. The stock solution was diluted with 70% methanol to yield a series of appropriate concentrations. All the prepared samples were stored at ‐10°C till their analysis.

### HPLC and Q‐TOF‐MS analysis conditions

HPLC analysis was carried out on an Agilent 1200 series equipped with a diode array detector (Agilent Technologies, USA). Chromatographic separation was performed on a C_18_ column (4.6 × 150 mm, 3.5 µm, Zorbax eclipse plus, Agilent USA) at 30°C with a guard column (4.6 × 12.5 mm, 5 µm, Zorbax eclipse plus). The mobile phase consisted of H_2_O (solvent A) and ACN (solvent B) with 0.1% (v/v) formic acid, respectively. The gradient program was as follows: 0‐19 min, 10‐46% B; 19‐20 min, 46‐10% B. A post‐run equilibrium time of 3 min was used for all samples. The flow rate was set at 1 mL/min with ultraviolet (UV) detection at 350 nm. The T splitter gave a flow rate of 0.25 mL/min toward the MS detector.

Mass spectrometry was performed using an Agilent 6540 Q‐TOF‐MS system (Agilent Corp., USA) equipped with a jet stream ESI interface. The TOF/MS system was operated in both negative and positive ion modes. Mass spectra were recorded over the mass range *m/z* 50‐1100. The mass analysis conditions were set as follows: drying gas (N_2_) flow rate, 10 L/min; nebulizer, 40 psi; drying gas temperature, 350°C; sheath gas temperature, 350°C; capillary voltage, 3500 V; fragmentor voltage, 120 V.

### Method validation for the determination of five flavonoids

The linearity, precision (inter‐day and intra‐day), repeatability, and recovery were carried out to validate the HPLC method according to ICH guideline (Validation of Analytical Procedures: Text and Methodology Q2 (R1), 2005)

Six working solutions were analyzed for the construction of calibration curves. Linearity was evaluated by the calculation of a regression line by the method of least squares. The limits of detection (LOD) and quantification (LOQ) were estimated experimentally by injecting a series of dilute solutions with known concentrations until the signal‐to‐noise ratio (S/N) for the standards reached a 3:1 ratio for LOD and 10:1 for LOQ, respectively. Intra‐ and inter‐day variations were applied to determine the precision of the developed method. The repeatability of the proposed HPLC method was studied at three levels (0.10 g, 0.20 g, 0.25 g) of the sample No.15 (Senegal). The samples of each level were extracted and analyzed triplicates. The accuracy of the method was tested by detecting the recovery, which was evaluated by adding three concentration levels (low, middle and high) of standard solutions into certain amount (0.10 g) of sample No.15 (Senegal) (Peng *et al*., 2013). The samples were extracted and analyzed for quantitative analysis as the developed method mentioned above.

### Data analysis

Accurate mass data were recorded and processed by MassHunter B.04.00 software (Agilent Technologies, USA). Principal components analysis (PCA) and similarity analysis (SA) were performed to analyze the 31 samples of *H. sabdariffa* L. based on the common characteristic peaks. PCA and SA were calculated and generated using a professional software named “ChemPattern 1.0.1.0” (fingerprint chromatography processing software, ChemMind Technologies (Beijing) Co., Ltd., China). All the data were pretreated including data normalization and chromatograms alignment before PCA and SA.

## Results and discussion

### Optimization of sample extraction and chromatographic conditions

According to the previous studies, methanol‐water (70:30) has a higher efficiency in extracting flavonoids than ethanol‐water (70:30) (Chen *et al*., 2012). Ultrasonic extraction is considered a simpler and more effective method for extraction of flavonoids in the plant leaves. Moreover, ultrasonication for 30 min gave a similar result as the soxhlet for 240 min (Zu *et al*., 2006). The ultrasonic extraction method in this study was also optimized including extraction times (30 min, 45 min and 60 min), extraction temperatures (room temperature, 35°C and 45°C) and times of extraction (once and twice). The peak areas of the five flavonoids were used as a marker for evaluation of extraction efficiency. There were no significant differences in the peak areas of the five flavonoids among different extraction conditions as described above. Therefore, the simple and convenient extraction method was selected as follows: solvent, 70% methanol; extraction temperature, room temperature; ultrasonic extraction time, 30 min.

**Figure 2:**
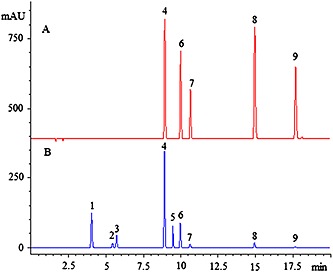
Chromatograms of mixed standards (A) including: rutin (4), kaempferol‐3‐o‐rutinoside (6), kaempferol‐3‐o‐glucoside (7), quercetin (8), kaempferol (9), and (B) common pattern of HPLC fingerprint of 31 samples of *H. sabdariffa* L.

HPLC conditions including chromatographic column, mobile phase and detection wavelength were optimized. The best results were obtained using an Agilent zorbax eclipse plus C_18_ (150 × 4.6 mm i.d., 3.5 µm) column at 30°C, with gradient elution of 0.1 % aqueous formic acid and ACN with 0.1% formic acid (v/v) as the mobile phase. According to the peak area of principal peaks, 350 nm was chosen as the detection wavelength. Under the optimized conditions, the separation of five marker compounds can be easily achieved in 20 min. Typical HPLC‐DAD chromatograms are shown in Fig. 2A.

### HPLC fingerprint analysis

**Figure 3:**
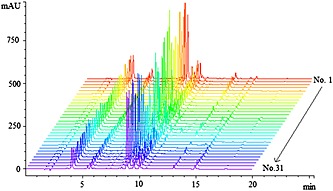
The HPLC fingerprints of the 31 *H. sabdariffa* L. samples at wavelength 350 nm.

The fingerprints of 31 samples of *H. sabdariffa* L. were obtained under the optimal HPLC conditions and are shown in Fig. 3. Peaks that existed in all the samples were assigned as “common pattern”, which was generated based on all chromatograms by the professional software of ChemPattern 1.0.1.0 (Fig. 2B).

HPLC fingerprint method precision and reproducibility were evaluated by the analysis of five runs of the same sample solution and five replicates from the same sample, respectively. The relative standard deviations (RSD) of retention time (RT) and peak area (PA) of 9 characteristic peaks in the precision test were found in the range of 0.15‐3.27%, whereas in the reproducibility test the RSDs of RT and PA were also below 0.75 and 4.26%, respectively. The stability of sample solution was evaluated at different time points (0, 2, 4, 8, 12 and 24 h), and the RSDs of RT and PA were less than 0.32 and 3.90%, respectively. All of these results indicated that the HPLC fingerprint method was reliable.

### Identification of major compounds in *H. sabdariffa* L.

The structural identification of 9 characteristic peaks was performed by the LC‐Q‐TOF‐MS. Both negative and positive ion modes were used because they provided more information about chemical structure. Some phenolic acids, flavonoids and ascorbic acid have been reported in the leaves of *H. sabdariffa* L. (Rodriguez‐Medina *et al*., 2009; Wang *et al*., 2014; Kumar *et al*., 2015)*.* In this study, the nine characteristic peaks were identified by comparison of their retention time and accurate MS with those of reference standards. The MS data are shown in Table 2. Of these 9 compounds identified, the 5 flavonoids (rutin, kaempferol‐3‐o‐rutinoside, kaempferol‐3‐o‐glucoside, quercetin and kaempferol) were chosen as the marked components.

**Table 2:** Identification of chemical constituents in *H. sabdariffa* L. by LC‐Q‐TOF‐MS

No.	RT (min)	[M + H]^+^	Main fragments	Measured *m*/*z*	Calculated *m*/*z*	Error (ppm)	Formula	Identification*
1.	4.05	355.1025	163.0390	354.0952	354.0951	‐0.33	C_16_H_18_O_9_	Neochlorogenic acid
2.	5.45	355.1020	163.0390	354.0947	354.0951	1.14	C_16_H_18_O_9_	Chlorogenic acid
3.	5.71	355.1029	163.0391	354.0956	354.0951	‐1.46	C_16_H_18_O_9_	Cryptochlorogenic acid
4	8.90	611.1607	465.1025, 303.0466	610.1526	610.1534	1.36	C_27_H_30_O_16_	Rutin
5	9.50	465.1033	303.0498	464.0960	464.0955	‐1.1	C_21_H_20_O_12_	Isoquercitin
6	10.01	595.1663	449.1075, 287.0553	594.1590	594.1585	‐0.86	C_27_H_30_O_15_	Kaempferol‐3‐o‐rutinoside
7	10.66	449.1070	287.0541	448.0997	448.1006	1.88	C_21_H_20_O_11_	Kaempferol‐3‐o‐glucoside
8	14.96	303.0495	158.0028	302.0422	302.0427	1.39	C_15_H_10_O_7_	Quercetin
9	17.67	287.0549	158.0029	286.0476	286.0477	0.46	C_15_H_10_O_6_	Kaempferol

* All the identified compounds were compared with standard compounds.

### Method validation for the determination of five flavonoids

The linear ranges, regression equations, LODs and LOQs of the five analytes were detected using the developed HPLC method. As shown in Table 3, the calibration curves of the analytes showed good linearity (*r* ^2^ ≥ 0.9996) with given concentration ranges. LOD and LOQ values were less than 0.10 µg/mL and 0.35 µg/mL, respectively, and showed the adequate sensitivity of the proposed method. The intra‐ and inter‐day variations (RSD) of five analytes peak areas were less than 0.67% and 0.90%, while retention time were less than 0.26% and 0.26%, respectively (Table 4). The average recoveries obtained in this study ranged from 86.80% to 103.50% (Table 5). The repeatability of the developed method was evaluated at three levels (Table 6). The results showed that the repeatability (RSD, n = 3) was less than 1.05% (0.10 g), 0.81% (0.20 g), 1.29% (0.25 g), respectively. Therefore, the proposed HPLC method could be considered accurate for quantitative determination of the five investigated compounds.

**Table 3:** Linearity, LOD and LOQ in the determination of analytes

Compounds	Linear range (µg/mL)	Regression equation^a^	Correlation coefficient *r* ^2^	LOD (µg/mL)	LOQ (µg/mL)
Rutin	2.65‐340.00	y = 14.832x + 1.400	0.9996	0.10	0.35
Kaempferol‐3‐o‐rutinoside	4.00‐122.00	y = 17.225x‐17.691	0.9997	0.10	0.32
Kaempferol‐3‐o‐glucoside	0.20‐50.00	y = 18.385x‐0.512	1.0000	0.07	0.20
Quercetin	0.40‐20.00	y = 25.426x‐0.847	0.9999	0.09	0.28
Kaempferol	0.20‐15.75	y = 35.377x‐0.172	0.9998	0.07	0.20

a y is the peak area; x is the concentration (µg/mL).

**Table 4:** Intra‐ and interday precision of the investigated analytes

Analytes	Concentration Level (ug/mL)	Intra‐day (RSD, %, n = 6)		Inter‐day (RSD, %, n = 3)	
		Retention time	Peak area	Retention time	Peak area
Rutin	3.4	0.26	0.18	0.17	0.21
	34.0	0.09	0.09	0.19	0.09
	170.0	0.06	0.07	0.26	0.32
Kaempferol‐3‐o‐rutinoside	2.0	0.26	0.29	0.11	0.24
	20.0	0.07	0.11	0.18	0.06
	100.0	0.06	0.06	0.23	0.32
Kaempferol‐3‐o‐glucoside	1.0	0.25	0.30	0.09	0.16
	10.0	0.07	0.10	0.18	0.05
	50.0	0.06	0.06	0.23	0.32
Quercetin	2.0	0.20	0.64	0.26	0.90
	20.0	0.08	0.12	0.13	0.45
	100.0	0.03	0.08	0.18	0.40
Kaempferol	1.0	0.17	0.67	0.03	0.37
	10.0	0.07	0.11	0.12	0.09
	50.0	0.04	0.06	0.16	0.27

**Table 5:** Recoveries for the assay of five compounds in *H. sabdariffa* L. leaves

Analytes	Original amount (µg)	Spiked (µg)	Amount found^a^ (µg)	Recovery^b^ (%)	RSD^c^ (%)
Rutin	683.05	347.70	1036.64	101.69	2.59
	683.05	695.40	1402.76	103.50	0.70
	683.05	1043.10	1752.19	102.50	1.50
Kaempferol‐3‐o‐rutinoside	888.67	940.00	1801.70	97.13	3.72
	888.67	1880.00	2696.76	96.18	1.94
	888.67	2820.00	3336.39	86.80	1.49
Kaempferol‐3‐o‐glucoside	194.46	220.00	410.85	98.36	5.04
	194.46	440.00	632.84	99.63	1.76
	194.46	660.00	774.56	87.89	1.94
Quercetin	35.33	31.10	66.44	100.03	1.68
	35.33	62.20	96.67	98.62	5.70
	35.33	93.30	128.97	100.36	1.58
Kaempferol	22.37	13.02	34.75	95.08	1.67
	22.37	26.04	46.82	93.89	3.01
	22.37	39.06	59.16	94.19	0.70

a The data was present as average of three determination.

b Recovery (%) = (amount found‐original amount)/spiked amount × 100.

c RSD (%) = (recovery SD/mean) × 100.

**Table 6:** Repeatability of five investigated analytes

Analytes	Repeatability (RSD, %, n = 3)		
	Level I (0.1 g of sample)	Level II (0.2 g of sample)	Level III (0.25 g of sample)
Rutin	0.58	0.81	0.46
Kaempferol‐3‐o‐rutinoside	0.84	0.65	0.41
Kaempferol‐3‐o‐glucoside	0.80	0.73	0.30
Quercetin	0.54	0.26	1.29
Kaempferol	1.05	0.54	0.53

### Quantitative analysis of five analytes in 31 *H. sabdariffa* L. accessions

The developed HPLC method was successfully applied to the simultaneous quantification of the five marker compounds in 31 accessions of *H. sabdariffa* L. The results are shown in Table 7. The quantitative analysis results showed that the content ranges (mg/g) were 0.47‐19.16 (rutin), 0.66‐8.65 (kaempferol‐3‐o‐rutinoside), 0.18‐1.94 (kaempferol‐3‐o‐glucoside), 0.18‐0.82 (quercetin), and 0.03‐0.22 (kaempferol), respectively. The total content of the five flavonoids showed great variations among different accessions, ranging from 3.35 to 23.30 mg/g. Among the tested samples, the sample No. 2 from Cuba had the highest contents of five flavonoids, while the sample No. 19 from USA had the lowest total amount (Table 7).

**Table 7:** Contents of the investigated compounds in the leaves of *H. sabdariffa* L.

No.^a^	Content, (mg/g, mean ± SD, n = 3)					
	Rutin	Kaempferol‐3‐o‐rutinoside	Kaempferol‐3‐o‐glucoside	Quercetin	Kaempferol	Total^b^
1	12.864 ± 0.230	2.860 ± 0.053	0.254 ± 0.029	0.333 ± 0.015	0.050 ± 0.001	16.361
2	18.810 ± 0.249	3.646 ± 0.067	0.355 ± 0.003	0.433 ± 0.005	0.062 ± 0.001	23.304
3	14.845 ± 0.174	3.448 ± 0.055	0.333 ± 0.003	0.277 ± 0.005	0.040 ± 0.001	18.943
4	11.182 ± 0.123	2.495 ± 0.023	0.228 ± 0.019	0.181 ± 0.017	0.027 ± 0.002	14.112
5	15.821 ± 0.467	4.069 ± 0.129	0.336 ± 0.029	0.305 ± 0.021	0.048 ± 0.003	20.579
6	9.988 ± 0.130	2.526 ± 0.040	0.270 ± 0.006	0.200 ± 0.005	0.032 ± 0.000	13.017
7	11.882 ± 0.165	2.809 ± 0.045	0.291 ± 0.009	0.224 ± 0.000	0.031 ± 0.000	15.237
8	9.820 ± 0.273	2.828 ± 0.082	0.344 ± 0.008	0.489 ± 0.011	0.083 ± 0.001	13.565
9	11.889 ± 0.201	2.277 ± 0.063	0.275 ± 0.002	0.471 ± 0.007	0.061 ± 0.001	14.974
10	19.164 ± 0.262	2.642 ± 0.043	0.269 ± 0.005	0.370 ± 0.009	0.038 ± 0.001	22.483
11	15.098 ± 0.510	2.654 ± 0.067	0.223 ± 0.010	0.380 ± 0.013	0.050 ± 0.001	18.406
12	9.765 ± 0.115	2.310 ± 0.016	0.208 ± 0.019	0.219 ± 0.008	0.032 ± 0.000	12.534
13	15.173 ± 0.441	2.177 ± 0.065	0.189 ± 0.003	0.394 ± 0.005	0.042 ± 0.000	17.976
14	12.259 ± 0.243	2.713 ± 0.052	0.354 ± 0.016	0.389 ± 0.015	0.057 ± 0.000	15.772
15	6.758 ± 0.020	8.654 ± 0.057	1.944 ± 0.014	0.347 ± 0.001	0.217 ± 0.001	17.920
16	11.075 ± 0.187	3.105 ± 0.056	0.713 ± 0.013	0.494 ± 0.013	0.077 ± 0.003	15.463
17	14.711 ± 0.295	2.844 ± 0.056	0.375 ± 0.011	0.397 ± 0.006	0.053 ± 0.001	18.381
18	10.490 ± 0.410	2.572 ± 0.103	0.255 ± 0.014	0.276 ± 0.015	0.043 ± 0.001	13.637
19	0.465 ± 0.002	0.661 ± 0.008	1.458 ± 0.012	0.693 ± 0.010	0.072 ± 0.001	3.349
20	10.089 ± 0.224	2.333 ± 0.041	0.206 ± 0.009	0.319 ± 0.008	0.052 ± 0.002	12.999
21	6.783 ± 0.258	1.721 ± 0.057	0.776 ± 0.032	0.426 ± 0.018	0.064 ± 0.002	9.770
22	7.050 ± 0.086	1.703 ± 0.049	0.773 ± 0.014	0.430 ± 0.008	0.062 ± 0.000	10.019
23	11.934 ± 0.125	2.553 ± 0.027	0.314 ± 0.022	0.371 ± 0.014	0.058 ± 0.001	15.229
24	14.123 ± 0.185	3.488 ± 0.027	0.478 ± 0.005	0.437 ± 0.002	0.079 ± 0.001	18.605
25	15.309 ± 0.202	2.202 ± 0.051	0.190 ± 0.020	0.563 ± 0.013	0.059 ± 0.001	18.322
26	15.979 ± 0.707	2.318 ± 0.057	0.203 ± 0.026	0.636 ± 0.018	0.066 ± 0.003	19.203
27	12.689 ± 0.358	1.881 ± 0.058	0.177 ± 0.002	0.508 ± 0.012	0.055 ± 0.002	15.309
28	16.067 ± 0.238	2.228 ± 0.052	0.232 ± 0.009	0.522 ± 0.012	0.051 ± 0.001	19.100
29	13.731 ± 0.266	1.870 ± 0.049	0.202 ± 0.016	0.520 ± 0.012	0.050 ± 0.000	16.374
30	15.151 ± 0.252	2.035 ± 0.054	0.199 ± 0.023	0.822 ± 0.008	0.079 ± 0.001	18.286
31	15.218 ± 0.082	2.241 ± 0.017	0.226 ± 0.014	0.544 ± 0.008	0.057 ± 0.001	18.286
Mean^c^	12.457	2.705	0.408	0.418	0.059	16.049
Min	0.465	0.661	0.177	0.181	0.027	3.349
Max	19.164	8.654	1.944	0.822	0.217	23.304

a Sample No. corresponds to Table 1.

b Total represents the sum of the individual selected five flavonoids.

c Mean, Min and Max were obtained based on the contents of 31 samples.

Based on the previous studies, it is found that both the total flavonoid content and antioxidant activity of *H. sabdariffa* L. leaves were higher than those of *H. sabdariffa* L. flowers (Chen *et al*., 2013; Mohd‐Esa *et al*., 2010). Variations in antioxidant activity may be due to different phenolic, flavonoid and ascorbic acid contents (Kumar *et al*., 2015). Therefore the total antioxidant activities of samples could vary greatly among different accessions of *H. sabdariffa* L.

### Principal component analysis (PCA)

**Figure 4:**
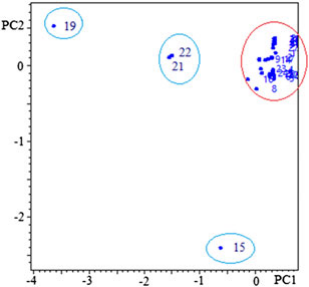
PC1‐PC2 score plot of 31 samples. The labels of the samples refer to Table 1.

To evaluate the variations among the 31 accessions, PCA was performed. The data of chromatographic fingerprints were imported into the ChemPattern 1.0.1.0 software. The score plot of the first two principal components (PC1‐PC2) is shown in Fig. 4.

The first two principal components (PC1‐PC2) were accounted for 85.9% of the total variance of the samples. The PCA analysis showed that the differences observed in the *H. sabdariffa* L. accessions were derived from the concentration of rutin. PC1, which explained 63.4% of the variance, was positively correlated with rutin content. For PC2 significant variables were flavonoid content and kaempferol‐3‐o‐rutinoside content.

PCA analysis revealed clearly the relationships among the tested samples. As shown in Fig. 4, the two samples No. 21 and 22 were clustered in one group, which were from Sudan. Except the sample No. 15 from Senegal and No. 19 from USA, the other samples can be clustered in one main group. The main group may be considered as rutin‐rich chemotype, which contains much more rutin than others. The results also indicated that samples No.15 and 19 produced greater variations in their chemical compositions and content. Because all the leaf samples were obtained from the accessions grown in the same place and under the same cultivation conditions, such variations may result from the inherent variability of the accessions. No. 19 could be a low quality accession in terms of its major flavonoid contents.

### Similarity analysis (SA)

**Figure 5:**
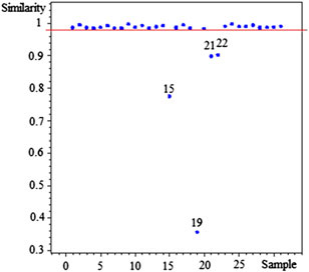
Similarity evaluation of HPLC fingerprint for *H. sabdariffa* L. Different samples (No.1‐31) are listed in Table 1.

Similarity analysis is a conventional method describing the similarity among the fingerprints. In this study, cosine similarity algorism was applied for the similarity analysis. By comparison with the fingerprint common pattern of *H. sabdariffa* L., the similarity index of 31 samples was not less than 0.983 except those of samples No. 15, 19, 21 and 22 (Fig. 5).

As shown in Fig. 5, taking 0.98 as the threshold, the samples with the correlation coefficients above it should be clustered to a group, which has been properly proved by the previous results from PCA. The different similarity values of samples means the different internal quality of these samples. Therefore, the developed HPLC fingerprint common pattern could be as a quality assessment model for classifying *H. sabdariffa* L. accessions.

Generally, the climatic and edaphic conditions are important environmental factors that can affect chemical composition of the leaf samples. In order to eliminate the environmental effect, we collected the seeds from 31 accessions and germinated and grew them in the same environment. The leaves were collected at the same time for quantitative and chemical fingerprint analyses. We assume the chemical variations among the accessions could be attributed to the variations of the seed sources where these individual accessions may have adapted to their original climatic and edaphic conditions. Therefore, the resulting genetic variations could be one of the key factors affecting the contents of bioactive compounds and quality of *H. sabdariffa* L. Many of these accessions could also be called ecotypes of the species. This study is a first comprehensive evaluation of the chemical variations among 31 *H. sabdariffa* L. accessions based on the combination of quantitative and chromatographic fingerprint analyses. Because of the tremendous potential of utilizing the leaves of this species for food, nutrition, and medicine, as well as value added products for human health, this research would be helpful for the quality control of *H. sabdariffa* L. in the future.

## Conclusion

A strategy for clarifying the chemical variations of different accessions of *H. sabdariffa* L. was developed. Firstly, chemical fingerprint analysis was performed based on the multivariate analysis methods (PCA and SA). Secondly, a simple HPLC method was developed and validated for simultaneously quantitative analysis of major flavonoids in 31 accessions of *H. sabdariffa* L. The samples were clustered in one group except four accessions (No.15, 19, 21 and 22). The chemical differences could be due to the inherent variability of the accessions. The proposed HPLC and fingerprint method were validated and proved to be reliable. The chemical fingerprint analysis is helpful to clarify the relationship among different accessions of *H. sabdariffa* L. and useful for quality control.
